# Human TRPA1 is a heat sensor displaying intrinsic U-shaped thermosensitivity

**DOI:** 10.1038/srep28763

**Published:** 2016-06-28

**Authors:** Lavanya Moparthi, Tatjana I. Kichko, Mirjam Eberhardt, Edward D. Högestätt, Per Kjellbom, Urban Johanson, Peter W. Reeh, Andreas Leffler, Milos R. Filipovic, Peter M. Zygmunt

**Affiliations:** 1 Department of Biochemistry and Structural Biology, Center for Molecular Protein Science, Lund University, SE-221 00 Lund, Sweden; 2 Institute of Physiology and Pathophysiology, Friedrich-Alexander University Erlangen-Nürnberg, Universitätsstrasse 17, 91054 Erlangen, Germany; 3 Department of Anesthesiology and Intensive Care, Hannover Medical School, Carl-Neuberg-Strasse 1, 30625 Hannover, Germany; 4 Clinical Chemistry and Pharmacology, Department of Laboratory Medicine, Lund University, SE-221 85 Lund, Sweden; 5 Department of Chemistry and Pharmacy, Friedrich-Alexander University Erlangen-Nuremberg, Egerlandstrasse 1, 91058 Erlangen, Germany; 6 IBGC, UMR 5095, Universite de Bordeaux, 1, rue Camille Saint Saëns, CS 61390, 33077 Bordeaux cedex, France

## Abstract

Thermosensitive Transient Receptor Potential (TRP) channels are believed to respond to either cold or heat. In the case of TRP subtype A1 (TRPA1), there seems to be a species-dependent divergence in temperature sensation as non-mammalian TRPA1 is heat-sensitive whereas mammalian TRPA1 is sensitive to cold. It has been speculated but never experimentally proven that TRPA1 and other temperature-sensitive ion channels have the inherent capability of responding to both cold and heat. Here we show that redox modification and ligands affect human TRPA1 (hTRPA1) cold and heat sensing properties in lipid bilayer and whole-cell patch-clamp recordings as well as heat-evoked TRPA1-dependent calcitonin gene-related peptide (CGRP) release from mouse trachea. Studies of purified hTRPA1 intrinsic tryptophan fluorescence, in the absence of lipid bilayer, consolidate hTRPA1 as an intrinsic bidirectional thermosensor that is modified by the redox state and ligands. Thus, the heat sensing property of TRPA1 is conserved in mammalians, in which TRPA1 may contribute to sensing warmth and uncomfortable heat in addition to noxious cold.

The discovery of TRP ion channels as molecular thermosensors has opened up new avenues for understanding how organisms monitor and adapt to environmental temperature. In contrast to the role of TRPA1 as a heat sensor in non-mammalian species, the temperature-sensitivity of mammalian TRPA1 and its role in thermosensation has been debated ever since TRPA1 was proposed as a noxious cold sensor in the mouse sensory nervous system^1^. We have recently shown that the purified hTRPA1 is intrinsically sensitive to noxious cold when inserted into lipid bilayers and studied with the patch-clamp technique^2^, adding direct molecular evidence to the many studies suggesting that mammalian TRPA1 plays a role in noxious cold sensation^3^. There is, however, no evidence that TRPA1 itself is a heat sensor in mammalians, although being involved in heat detection^4^,^5^,^6^,^7^,^8^,^9^,^10^,^11^. It has been speculated that thermosensitive TRP channels are capable of sensing both cold and heat, but experimental evidence is lacking to support such a TRP channel U-shaped thermosensitivity^12^,^13^,^14^,^15^,^16^,^17^. Here we show that TRPA1 heat sensitivity is conserved in mammalians and for the first time provide experimental evidence of TRP channel inherent U-shaped thermosensitivity.

## Results and Discussion

**Figure 1:**
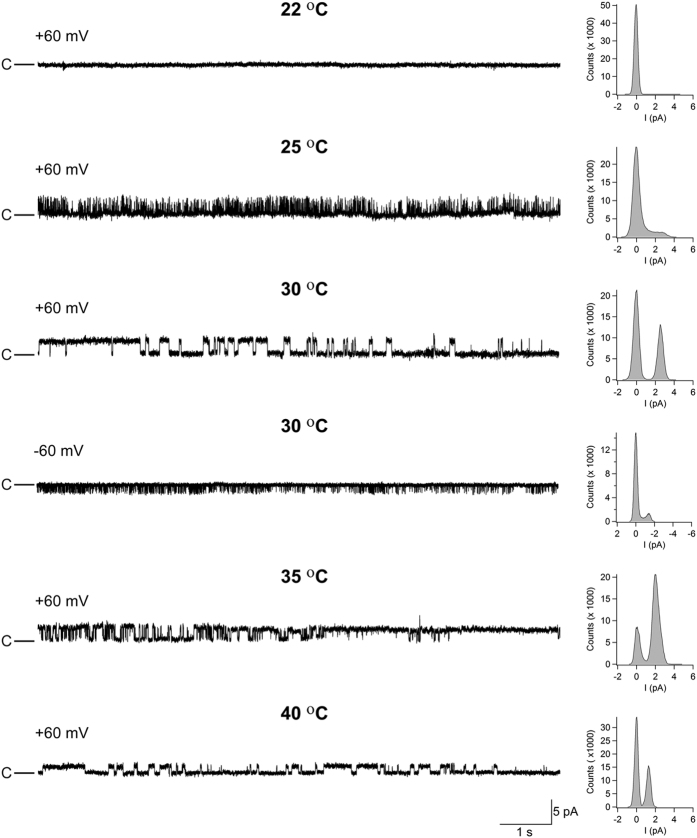
The purified hTRPA1 is a warmth receptor. Temperatures above 22 °C evoked steady state outward and inward hTRPA1 single channel currents at a test potential of +60 and −60 mV as shown by representative traces and the corresponding amplitude histograms. Purified hTRPA1 was inserted into planar lipid bilayers and channel currents were recorded with the patch-clamp technique in a symmetrical K^+^ solution (c indicates the closed channel state).

**Figure 2:**
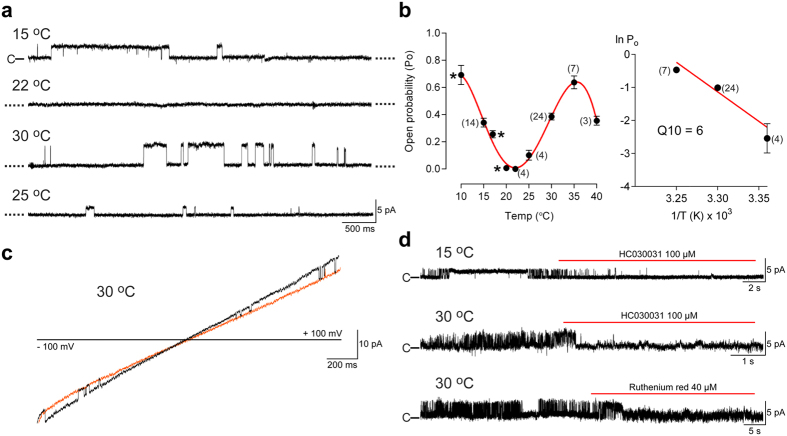
The purified hTRPA1 displays intrinsic U-shaped thermosensitivity. (**a**) Traces are part of a continuous recording (13 min) of hTRPA1 single channel currents at various temperatures at a test potential of +60 mV. (**b**) Left graph shows single-channel mean open probability values at different temperatures. Data points marked by asterisk were published previously^2^. Right graph shows Arrhenius plot for the heat responses (25, 30 and 35 °C). Data were obtained at a steady state test potential of +60 mV (see Fig. 1) and analysed only for a first exposure to the indicated temperatures. Data are represented as mean ± s.e.m. (numbers of experiments within parentheses). (**c**) Black trace shows hTRPA1 channel openings when separated from orange trace (baseline) at both negative and positive test potentials (2-s voltage ramps from −100 to +100 mV). (**d**) The selective TRPA1 antagonist HC030031 and the non-selective TRP channel pore blocker ruthenium red inhibited cold and heat hTRPA1 responses at a test potential of +60 mV (n = 3). Purified hTRPA1 was inserted into planar lipid bilayers and channel currents were recorded with the patch-clamp technique in a symmetrical K^+^ solution (c indicates the closed channel state).

The purified hTRPA1 inserted into lipid-bilayers responded with single-channel activity when exposed to increasing temperatures from 22 °C to 40 °C, and as previously reported^2^ to noxious cold (Figs 1 and 2, Table 1). Based on the single-channel mean open probability (Po) (Fig. 2b), we calculated a Q10 value of 6 from the Arrhenius plot (25 °C to 35 °C), which is close to the Q10 value 7.5 of the heterologously expressed TRPM3, a recently identified heat-activated TRP ion channel present in capsaicin-sensitive primary afferents^18^. At 40 °C, there was still substantial channel activity although Po decreased, possibly indicating channel gating desensitization (Fig. 2b). The single-channel mean conductance (Gs) did not increase with increasing temperatures (Table 1), suggesting that the TRPA1 channel pore is negatively affected by heat. As shown at 30 °C, hTRPA1 channel currents were observed at both positive and negative test potentials (Figs 1 and 2c), and the non-selective TRP channel pore blocker ruthenium red and the selective TRPA1 antagonist HC030031 inhibited temperature responses (Fig. 2d) without affecting bilayers (Supplementary Fig. 1). No currents were detected in bilayers without hTRPA1 when exposed to the same test temperatures (Supplementary Fig. 2).

**Figure 3:**
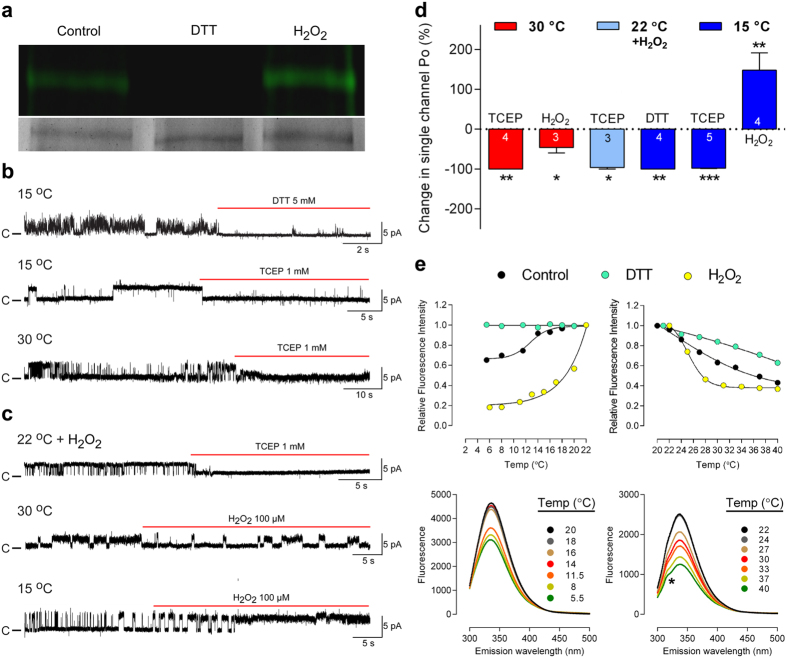
Redox modification influences the responsiveness of purified hTRPA1 to cold and heat. (**a**) The Cy3-dye disulphide labeling fluorescence assay (top gel) revealed that the purified hTRPA1 used for functional studies was partially oxidized (control) as reducing (DTT, 1 mM) and oxidizing (H_2_O_2_, 100 μM) agents abolished and increased the disulphide bond formation, respectively. Coomassie blue staining (bottom gel) shows the amount of protein used for analysis of respective treatment. (**b**) Cold and heat responses of hTRPA1 were abolished by the reducing agents DTT and TCEP. (**c**) H_2_O_2_ (100 μM) activated hTRPA1 at 22 °C (see also Table 1) and the activity was abolished with TCEP. Notably, H_2_O_2_ reduced heat and increased cold responses. (**b**,**c**) Purified hTRPA1 was inserted into planar lipid bilayers and channel currents were recorded with the patch-clamp technique in a symmetrical K^+^ solution at a test potential of +60 mV (c indicates the closed channel state). (**d**) Summary of the effect of DTT (5 mM), TCEP (1 mM) and H_2_O_2_ (100 μM) on hTRPA1 single channel mean open probability (Po) examined at 30 °C and 15 °C at a test potential of +60 mV. Shown is also the effect of TCEP (1 mM) on hTRPA1 single channel activity evoked by H_2_O_2_ (100 μM) at 22 °C at a test potential of +60 mV. Data are represented as mean ± s.e.m. of paired comparisons before and after treatment (numbers of experiments are shown within bars). *P < 0.05, **P < 0.01 and ***P < 0.001 indicate statistically significant differences using the Student’s paired t test. (**e**) As shown by analysis of hTRPA1 intrinsic tryptophan fluorescence, cold and heat produced lipid bilayer-independent conformational changes of hTRPA1 (control) that were potentiated and inhibited by H_2_O_2_ (100 μM) and DTT (500 μM), respectively (n = 3). The hump (*) was only observed at temperatures above 22 °C. The fluorescence intensity, emitted at 335 nm, for each indicated temperature was related to that of 22 °C and expressed as Relative Fluorescence Intensity. Data are represented as mean ± s.e.m. Spectra of control is shown below.

Because TRPA1 with its many cysteines is highly sensitive to thiol reactive agents including oxidants^3^, we asked if changes in redox state could affect the temperature sensitivity of hTRPA1, possibly explaining the many contradictory findings on mammalian TRPA1 and cold^3^ as well as the lack of mammalian TRPA1 heat responses in heterologous expression systems^19^,^20^,^21^. As shown by the Cy3-dye disulphide labeling assay, which has been used to study TRPA1 disulphide bond formation^22^, the purified hTRPA1 used for the bilayer patch-clamp recordings was partially oxidized, a condition that could be rectified by the thiol reducing agent dithiothreitol (DTT) and further oxidized by H_2_O_2_ (Fig. 3a). The consequences of changes in cysteine redox state for hTRPA1 channel activity are shown in Fig. 3b–d, where the reducing agents DTT and Tris (2-carboxy ethyl) phosphine (TCEP) inhibited cold and heat responses, and H_2_O_2_ evoked hTRPA1 channel activity at 22 °C that was blocked by TCEP. Furthermore, H_2_O_2_ decreased hTRPA1 channel activity triggered by 30 °C, and increased cold responses at 15 °C (Fig. 3c,d), possibly indicating different conformational states/gating mechanisms behind hTRPA1 heat and cold responses. Bilayers without hTRPA1 were unaffected by DTT, TCEP and H_2_O_2_ (Supplementary Fig. 1). The effects of these thiol modifying agents were studied at concentrations frequently used to explore TRP ion channel redox sensitivity without cytotoxic effects^23^,^24^,^25^,^26^,^27^. Taken together, the redox state of purified hTRPA1 determines its cold- and heat responsiveness.

As TRPA1 is a potential mechanosensor^28^, possibly indirectly affected by mechanical forces within the lipid bilayer due to temperature changes, we used the tryptophan fluorescence assay to study lipid bilayer-independent conformational changes of hTRPA1 when exposed to cold and warm temperatures, and also when treated with either DTT or H_2_O_2_ (Fig. 3e). Both cold and warm temperatures evoked changes in tryptophan fluorescence, supporting that hTRPA1 is intrinsically cold- and heat-sensitive. Notably, the non-identical change in hTRPA1 fluorescence between heating and cooling (measured at 335 nm) as well as the hump on fluorescence traces at lower emission wavelengths for hTRPA1 when exposed to warm temperatures (Fig. 3e), indicate that different hTRPA1 channel conformations are involved in its cold and heat sensation. The changes in tryptophan fluorescence were inhibited by reducing agents and enhanced by H_2_O_2_ pre-treatment, clearly showing that the inherent thermosensitivity of hTRPA1 is affected by its redox environment.

**Figure 4:**
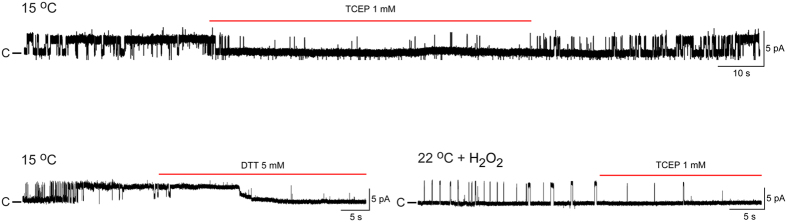
Redox modification influences the activity of purified hTRPA1 without its N-terminal ankyrin repeat domain (Δ1-688 hTRPA1). Representative traces show single channel activity for Δ1-688 hTRPA1 when inserted into planar lipid bilayers and exposed to cold (15 °C) or the oxidant H_2_O_2_ (100 μM) at 22 °C. The cold-evoked channel activity was inhibited by the reducing agents TCEP (1 mM, n = 4) and DTT (5 mM, n = 3), and as shown with TCEP the effect was reversible. The single channel mean open probability (Po) and conductance (Gs) values for cold were 0.46 ± 0.05 and 31 ± 6 pS, respectively (n = 6). The Po and Gs values for H_2_O_2_ were 0.16 ± 0.05 and 57 ± 8 pS, respectively (n = 6). The channel activity evoked by H_2_O_2_ was inhibited by TCEP (1 mM, n = 3). Channel currents were recorded with the patch-clamp technique a test potential of +60 mV in a symmetrical K^+^ solution (c indicates the closed channel state).

We have previously shown that the N-terminal ankyrin repeat domain is not needed for hTRPA1 to respond to cold^2^. In the present study, we found that a change in channel redox state by reducing agents inhibited N-terminal ankyrin repeat domain deleted hTRPA1 (Δ1-688 hTRPA1) cold responses (Fig. 4), suggesting that disulphide formation outside the N-terminal ankyrin repeat domain, which contains several redox sensitive cysteines^3^, also contribute to mammalian TRPA1 thermosensitivity. Similar to intact hTRPA1, H_2_O_2_ triggered Δ1-688 hTRPA1 channel activity at 22 °C that was inhibited by TCEP (Fig. 4). However, H_2_O_2_ evoked substantially less Δ1-688 hTRPA1 channel activity (Fig. 4) compared to hTRPA1 (Table 1), which is in line with the general view that cysteines within the N-terminal ankyrin repeat domain play an important role in oxidative activation of TRPA1^3^.

**Figure 5:**
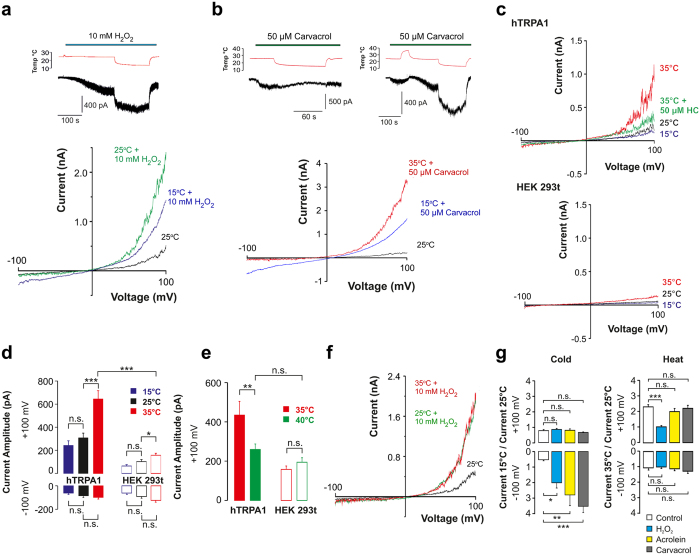
The cold and heat sensitivity of hTRPA1 is influenced by the redox state and ligands. (**a**) H_2_O_2_ triggered cold-evoked inward currents at −60 mV and in 500 ms voltage ramps (n = 12). (**b**) Carvacrol also triggered hTRPA1 cold responses, but only in cells pre-exposed to heat (n = 5–8). (**c,d**) Increasing the temperature from 25 °C to 35 °C evoked heterologously expressed hTRPA1 outward currents in HEK293t cells that were blocked by the selective TRPA1 antagonist HC030031 (91 ± 5%, n = 5), whereas only minor currents were observed in non-transfected cells (n = 10–19). (**e**) At 40 °C, outward currents were not different from those in non-transfected cells (n = 6–8). (**f**) H_2_O_2_, but not acrolein or carvacrol (see Supplementary Fig. 5), prevented a further rise in outward currents when increasing the temperature from 25 °C to 35 °C. (**g**) Bar graph summarizing the effect of cold and heat in the absence (control) and presence of the various treatments at either −100 or +100 mV (n = 5–7). Data are represented as mean ± s.e.m. *P < 0.05, **P < 0.01 and ***P < 0.001 indicate statistically significant differences using ANOVA and Tukey’s honest significant difference test.

In line with reports questioning hTRPA1 being a cold sensor^19^,^29^,^30^,^31^, we could not observe cold-induced hTRPA1 currents in HEK293t cells, using external Ca^2+^-free conditions to avoid rapid desensitization of hTRPA1, whereas the non-electrophilic TRPA1 activator carvacrol produced robust hTRPA1 currents at 25 °C, confirming that the channel was functionally expressed (Supplementary Fig. 3). However, pre-exposure of hTRPA1 to H_2_O_2_ or acrolein at threshold concentrations triggered cold-evoked currents (Fig. 5a,g, and Supplementary Fig. 3). Likewise, allyl isothiocyanate (AITC) disclosed the cold-sensitivity of hTRPA1 (Supplementary Fig. 3), which is in accordance with the finding that it potentiated rat TRPA1 cold responses^32^. A similar approach with carvacrol also sensitized hTRPA1 to cold, but only after having been exposed to heat (Fig. 5b), indicating that non-thiol reactive TRPA1 modulators can produce additional conformational changes of hTRPA1 allowing it to respond to changes in temperature. Indeed, the tryptophan fluorescence assay disclosed substantial hTRPA1 conformational changes to cold as well as heat when exposed to carvacrol (Supplementary Fig. 4). In contrast to cold, heat alone evoked hTRPA1 currents, which were reduced at 40 °C (Fig. 5c–e,g). An increase in temperature from 25 °C to 35 °C evoked larger currents in the presence of acrolein and carvacrol (see Supplementary Fig. 5) but not H_2_O_2_ (Fig. 5f,g), suggesting that heat responses were partially redox sensitive.

**Figure 6:**
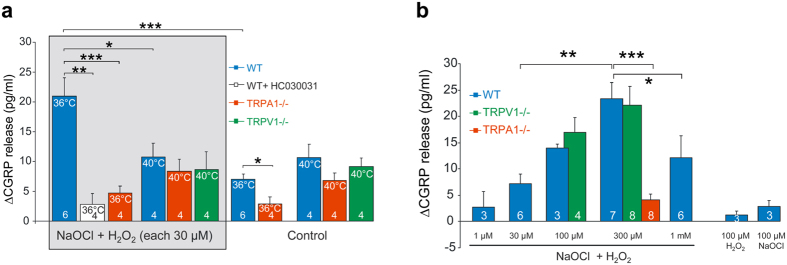
Mouse TRPA1 heat sensitivity is influenced by the redox state. (**a**) Raising the temperature from 22 °C to 36 °C caused a small TRPA1-dependent calcitonin gene-related peptide (CGRP) release from mouse trachea. After pre-incubation of trachea with subliminal concentrations of H_2_O_2_ + NaOCl, producing singlet oxygen, there was a substantial increase in CGRP release at 36 °C that was inhibited by the selective TRPA1 antagonist HC030031 (50 μM) and in TRPA1 knock-out mice. (**b**) At 37 °C, acute exposure to various concentrations of H_2_O_2_ and NaOCl combined caused a concentration-dependent release of CGRP that was dependent on TRPA1 but not TRPV1. Under these conditions, H_2_O_2_ and NaOCl (each at 100 μM) alone evoked minor CGRP release compared to their combination. Interestingly, the highest concentration of H_2_O_2_ and NaOCl combined (each 1 mM) was less effective causing CGRP release possibly due to desensitization of TRPA1. Data are represented as mean ± s.e.m. (numbers of experiments within bars). *P < 0.05, **P < 0.01 and ***P < 0.001 indicate statistically significant differences using ANOVA Tukey’s honest significant difference Tukey’s honest significant difference test.

To study TRPA1 in its native environment, we used the heat-sensitive mouse trachea preparation, measuring stimulated CGRP release from sensory neurons as a physiological readout of TRPA1 and possibly TRPV1 activity^33^. We observed a robust TRPA1-dependent CGRP release upon warming (to 36 °C) under oxidative stress, achieved by combining H_2_O_2_ and NaOCl to produce the strong oxidant singlet oxygen^34^ (Fig. 6 and Supplementary Fig. 6). Although, recombinant TRPV1 can be sensitized to heat by oxidizing agents^35^, and the mouse trachea treated with bradykinin (not oxidation) showed an increased TRPV1-mediated heat response at the temperatures (22–40 °C) studied here^33^, we did not find any contribution of TRPV1 to heat-evoked CGRP release (Fig. 6). The classical oxidants H_2_O_2_ and NaOCl each alone were ineffective (Fig. 6b), most likely because they dissociate in aqueous solutions and become charged, in contrast to singlet oxygen, thereby having difficulties diffusing through intact tissues such as the mouse trachea and to gain access to intracellular TRPA1 cysteine thiols. A small but significant TRPA1-mediated CGRP release was also detected in the absence of chemical oxidation (Fig. 6a). Thus, the mouse TRPA1 is not only sensitive to heat after single point mutations within its N-terminal ARD^21^, but also as naïve and in its native environment depending on the cellular redox state, and without the influence of TRPV1, which in most studies is thought to determine TRPA1-mediated heat responses^4^,^5^,^6^,^7^,^8^,^9^,^10^,^11^.

The increase in temperature to 40 °C caused a smaller TRPA1/TRPV1-independent CGRP release in the mouse trachea (Fig. 6a), which is in line with the reduced hTRPA1 activity observed at 40 °C in the bilayer and whole-cell patch clamp recordings (Figs 2b and 5e). A reduced mammalian TRPA1 activity was also reported when the temperature was increased slightly above body temperature^20^. Even though the activity of mammalian TRPA1 declines when exposed to noxious heat *in vitro*, it does not exclude TRPA1 from having a physiological role as a sensor of warm and hot temperatures. For example, TRPA1 has been shown to define the temperature threshold for noxious heat in mice^11^, and TRPA1 sensitization may contribute to heat hyperalgesia in animals and humans^4^,^5^,^6^,^7^,^8^,^9^,^10^. It is also possible that TRPA1 as a redox sensitive heat detector triggers itch and is involved in sensory nerve-mediated blood flow regulation in the skin^36^,^37^,^38^.

Numerous studies have attempted to pinpoint the molecular temperature sensing structure(s) in TRPA1 and other thermosensitive TRP channels, and various concepts have been put forward to explain how changes in temperature causes channel activation^12^,^13^,^14^,^15^,^16^,^17^,^39^. In this study, we provide for the first time experimental evidence of inherent TRP channel U-shaped thermosensitivity using the purified hTRPA1. Whether the ability of TRPA1 to directly sense both cold and heat is shared by other thermo TRP channels, and can be explained by the heat capacity model or allosteric coupling of different thermosensor modules to channel gating^12^,^13^,^14^,^17^ remains to be investigated.

In conclusion, the TRPA1 heat-sensitivity is conserved in mammalians and its inherent U-shaped thermosensitivity is influenced by the redox state and ligands. The responsiveness of primary afferents to innocuous heat is dependent on TRPA1 and its redox environment, suggesting a physiological role of TRPA1 as a heat sensor in mammalians. Although we cannot provide a final mechanistic insight into temperature gating of TRPA1, our findings indicate that different TRPA1 channel conformations are involved in its cold and heat sensation. Further studies of purified hTRPA1 may help to unravel the molecular mechanisms linking both cold and warm temperatures to channel gating in TRP and other ion channels.

## Methods

### Recording of hTRPA1 activity in planar lipid bilayers

The expression and purification of hTRPA1 and N-terminal ARD deleted hTRPA1 (Δ1-688 hTRPA1) were performed as described previously^2^ except that the β-mercaptoethanol was omitted from the wash and elution buffers used in the purification of Δ1-688 hTRPA1. Detergent purified hTRPA1 and Δ1-688 hTRPA1 were reconstituted either into preformed planar bilayers, or giant unilamellar vesicles (GUVs)^2^. Briefly, planar lipid bilayers were formed by pipetting 5 μl of either empty GUVs or protein reconstituted into GUVs on patch-clamp chips (1–2 μm, 3.5–5 MΩ resistance) which were mounted on a recording chamber. Following giga Ohm seal formation, single channel activity was recorded using the Port-a-Patch (Nanion Technologies) at both positive and negative test potentials in a symmetrical K^+^ solution adjusted to pH 7.2 with KOH and containing (in mM): 50 KCl, 10 NaCl, 60 KF, 20 EGTA, and 10 HEPES. The patch-clamp experiments were performed at various temperatures, for which the Port-a-Patch was equipped with an external perfusion system (Nanion Technologies) and an SC-20 dual in-line solution cooler/heater connected to a temperature controlled (CL-100) liquid cooling system (Warner Instruments). Signals were acquired with an EPC 10 amplifier (HEKA) and the data acquisition software Patchmaster (HEKA) at a sampling rate of 50 kHz. The recorded data were digitally filtered at 3 kHz. Electrophysiological data were analysed using Clampfit 9 (Molecular Devices) and Igor Pro (Wave Metrics software). Data were filtered at 1,000 and 500 Hz low-pass Gaussian filter for analysis and traces, respectively. The single-channel mean conductance (Gs) was obtained from a Gaussian fit of all-points amplitude histograms. The single-channel mean open probability (Po) was calculated from time constant values, which were obtained from exponential standard fits of dwell time histograms. The Q10 values were obtained from the slope of regression line fitted to the Arrhenius plot^40^,^41^. The Student’s paired t test was used for statistical analysis and P < 0.05 was considered statistically significant. Data points represent means ± s.e.m. of the given number (n) of experiments.

### Cy3-dye labelling of protein disulphides using modified biotin-switch

Purified hTRPA1 was treated with 0.1 mM H_2_O_2_ or 1 mM DTT for 30 min at 37 °C. Protein was precipitated with chloroform/methanol/water (1/4/4), dried under argon and re-suspended in PBS containing 1.5% SDS. Free thiols were blocked with N-ethyl maleimide (1 mM, 1 h at 37 °C) and oxidized cysteines reduced with 10 mM DTT (1 h at 37 °C) in the following step. Protein was again precipitated using chloroform/methanol/water protocol and re-suspended in PBS containing 1.5% SDS and 0.1 mM Cy3-maleimide. Samples were incubated 1 h at 37 °C, resolved in 10% SDS electrophoresis and visualized by ChemiDoc (BioRad) imaging system.

### Intrinsic tryptophan fluorescence assay

Conformational changes in hTRPA1 were recorded on FP-8200 spectrofluorometer (Jasco, Germany) with thermostat unit. Protein was incubated at each desired temperature for 15 min and emission spectra recorded using excitation wavelength of 280 nm. For H_2_O_2_ treatments, in order to achieve the maximal thiol oxidation, the protein was mixed with H_2_O_2_ and left overnight and the spectra recorded the following day. Cold and heat experiments were done in separate experiments and for better presentation the spectra are normalized to the starting spectrum at room temperature. DTT and carvacrol treatments were performed prior the measurements and samples incubated for 1 h to achieve stable modification. To keep the same oxygen levels in all samples and to prevent the spontaneous protein aggregation/precipitation, the measurements were performed in the sealed anaerobic cuvettes with constant stirring.

### Whole-cell patch-clamp recordings

HEK293 were cultured in standard DMEM (D-MEM, Gibco, BRL Life Technologies, Karlsruhe Germany) with 10% FBS (Biochrom, Berlin Germany), 100 U/ml penicillin and 100 μg/ml streptomycin (Gibco, Karlsruhe, Germany) and 2 mM Glutamax (Gibco, Karlsruhe Germany). They were transfected with plasmids of hTRPA1 and eGFP to visualize transfected cells using jetPEI transfection reagent according to the instructions of the manufacturer (Polyplus-Transfection, France). Cells were split into 35 mm dishes, cultured at 37 °C and 5% CO_2_ and patch clamped 24 to 48 h after transfection. For some experiments naïve HEK 293t cells were used as a control.

Voltage-clamp patch clamp experiments were carried out with a HEKA Electronics USB 10 amplifier and the Patchmaster Software (HEKA Electronics, Lambrecht, Germany) installed on a conventional PC. Borosilicate pipettes with a resistance of 1–2 MΩ were filled with a pipette solution containing [mM]: KCl 140, MgCl_2_ 2, EGTA 5, HEPES 10 with pH adjusted to 7.4 by KOH. Standard calcium free extracellular solution contained in [mM]: NaCl 140, KCl 5, MgCl_2_ 2, EGTA 5, HEPES 10 and glucose 10. pH 7.4 was adjusted by TMA-OH. For TRP channel mediated membrane currents, data were sampled at 10 kHz and filtered at 2 kHz. Cells were either constantly held at a membrane potential of −60 mV or 500 ms long voltage ramps from −100 to +100 mV were applied repetitively.

All substances were applied by a gravity-driven perfusion system allowing a focal application of the test solution within a distance of <100 μm from the cell. Temperature of the applied test solutions was controlled with a perfusion system incorporating a rapid-feedback temperature control allowing rapid heating or cooling^42^. The Patchmaster software (HEKA Elektronik) was used to pass current to an insulated copper wire coiled around the capillary tip of the outlet of the perfusion system. Test solutions were precooled by ice cold water, and for application of cold stimuli the continuous current necessary to warm solutions to room temperature was reduced. Heat stimuli were applied by increasing current to the coiled wire at the capillary tip. The temperature of the test solution was constantly measured using a miniature thermocouple fixed at the orifice of the capillary tip. The Patchmaster/Fitmaster software (HEKA Elektronik) was used for acquisition of temperature and current data and for off-line analysis. One-way ANOVA followed by the Tukey’s honest significant difference test was used for statistical analysis and P < 0.05 was considered statistically significant. Data points represent means ± s.e.m. of the given number (n) of experiments.

### Calcitonin gene-related peptide release

The experiments were carried out in accordance with the guidelines of the International Association for the Study of Pain^43^ and approved by the Animal Authority of the District Government of Mittelfranken (Ansbach, Germany). Adult C57BL/6, TRPV1−/− and TRPA1−/− mice were used. Breeding pairs of heterozygous TRPV1 and TRPA1 mutants were obtained from Dr. John Davis^44^ and Dr. David Corey^45^ and continuously backcrossed to C57BL/6. The mice were housed in group cages in a temperature-controlled environment on a 12 h light/dark cycle and were supplied with water and food ad libitum. Mice of either sex (body weight 15–25 g) were sacrificed by exposure to a rising CO_2_ concentration (approved by the Animal Protection Authority, District Government of Mittelfranken, Ansbach, Germany).

The trachea was excised together with the two main bronchi and hemisected along the sagittal midline. One half of the bronchotracheal preparation was used as control and the other half for experimental treatments (here oxidant pretreatment), taking advantage of the lesser intraindividual than interindividual variability of CGRP release. The preparations were first placed for 30 min at 37 °C in carbogen-gassed (95% O_2_, 5% CO_2_, obtaining pH 7.4) synthetic interstitial fluid (SIF) containing (in mM) 107.8 NaCl, 3.5 KCl, 1.53 CaCl_2_, 0.69 MgSO_4_, 26.2 NaHCO_3_, 1.67 NaH_2_PO_4_, 9.64 sodium gluconate. After the initial rest period, the isolated trachea was consecutively incubated for 5 min in each of four test tubes containing 125 μl SIF and mounted in a shaking bath at 22 °C. The first incubation was to determine basal CGRP release, the second through fourth tube did or did not contain the two oxidants NaOCl and H_2_O_2_ (both 30 μM) diluted in SIF. However, the fourth tube was placed in a shaking bath at either 36 ° or 40 °C to stimulate the tracheal nerve endings. The fifth incubation was again in SIF at 22 °C to determine the recovery from stimulation. To establish the concentration-response relationship of CGRP release evoked by NaOCl + H_2_O_2_, four incubations of trachea preparations were performed at constant 37 °C, the third one containing both oxidants at various equimolar concentrations or, for control, either NaOCl or H_2_O_2_ at 100 μM concentration.

To apply the oxidants, concentrated stock solutions of NaOCl and H_2_O_2_ were freshly prepared and minute volumes were synchronously pipetted into the incubation fluid as soon as the trachea preparation was immersed.

CGRP enzyme immunoassay (EIA): The CGRP content of the incubation fluid was measured using commercial enzyme immunoassay (EIA) kits with a detection threshold of 5 pg/ml (Bertin Pharma, Montigny-le-Bretonneux, France). For this purpose 100 μl sample fluid were stored on ice and mixed, immediately following the incubation, with 25 μl of fivefold-concentrated commercial CGRP-EIA buffer (Bertin) that contained a proprietary cocktail of peptidase inhibitors. The CGRP-EIA procedures were run after the end of the experiment; the antibody reactions took place overnight. The EIA plates were determined photometrically using a microplate reader (Dynatech, Channel Islands, UK). All results are processed as measured by the EIA in pg CGRP/ml SIF. Reducing interindividual variability and day-to-day baseline variability, the data were referred to the third (or second) individual baseline value (before stimulation). This value was subtracted from all five (or four) data points of a typical experiment so that only the absolute change in CGRP release (∆ pg/ml) is displayed in the figures.

Statistical comparisons were performed using Statistica 7 software (Statsoft, Tulsa, USA). All time series of experimental values were first analyzed for the effect of stimulation as compared to baseline using the nonparametric Wilcoxon matched pairs test. The baseline-corrected (i.e., ∆ pg/ml) CGRP values were entered into a one-way analysis of variance (ANOVA) followed by Tukey’s honest significant difference test, focusing on the peak values of stimulated CGRP release. P < 0.05 was considered statistically significant. Data points represent means ± s.e.m. of the given number (n) of experiments.

## Additional Information

**How to cite this article**: Moparthi, L. *et al*. Human TRPA1 is a heat sensor displaying intrinsic U-shaped thermosensitivity. *Sci. Rep*. **6**, 28763; doi: 10.1038/srep28763 (2016).

## Supplementary Material

Supplementary Information

## Figures and Tables

**Table 1:** Single channel mean open probability and conductance values for hTRPA1.

Stimuli	Voltage (mV)	Open probability (mean ± SEM)	n	Conductance (pS) (mean ± SEM)	n
40 °C	+60	0.35 ± 0.03	3	26 ± 2	3
35 °C	+60	0.64 ± 0.05	7	36 ± 5	6
30 °C	+60	0.38 ± 0.02	24	36 ± 3	22
30 °C	−60	0.13 ± 0.03	6	31 ± 5	6
25 °C	+60	0.10 ± 0.04	4	35 ± 4	3
22 °C	+60	0.00 ± 0.00	4	0 ± 0	4
22 °C + H_2_O_2_ (100 μM)	+60	0.57 ± 0.06	7	63 ± 9	7
15 °C	+60	0.34 ± 0.03	10	43 ± 3	7
