# Supplementary material for: Human TRPA1 is a heat sensor displaying intrinsic U-shaped thermosensitivity
Source: Sci Rep. 2016 Jun 28;6:28763. doi: 10.1038/srep28763 (PMC4923899; doi:10.1038/srep28763)
Supplement: Supplementary Information [file srep28763-s1.pdf]

Human TRPA1 is a heat sensor displaying intrinsic U-shaped thermosensitivity

Lavanya Moparthi, Tatjana I. Kichko, Mirjam Eberhardt, Edward D. Högestätt, Per Kjellbom, Urban Johanson, Peter W. Reeh, Andreas Leffler, Milos R. Filipovic and Peter M. Zygmunt

Supplementary Information

Figures 1-6

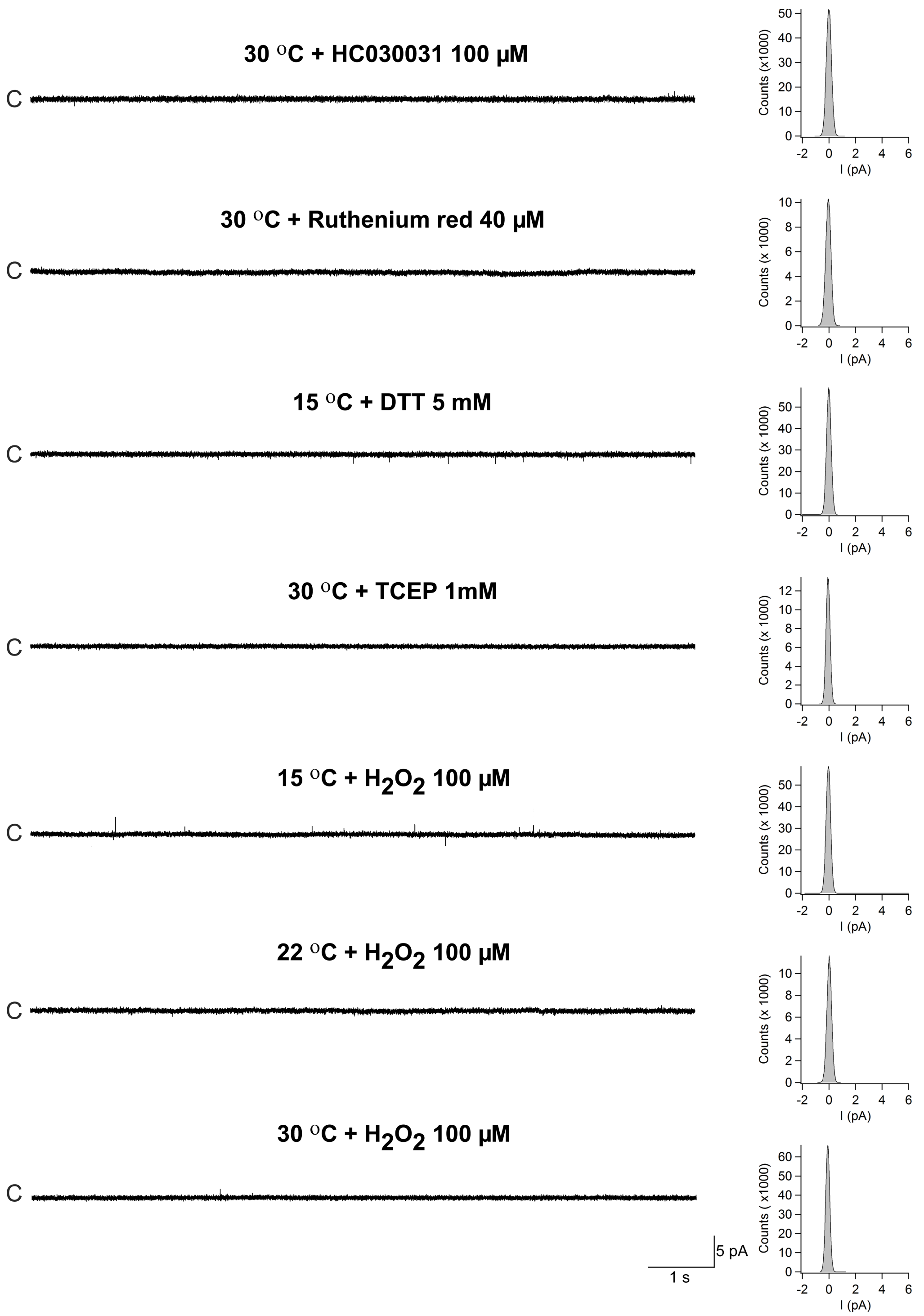

Supplementary Fig. 1. The TRPA1 antagonists HC030031 and ruthenium red as well as reducing (DTT and TCEP) and oxidizing (H_2O_2) agents had no effects on lipid bilayers without hTRPA1 at a test potential of +60 mV (n = 3-4). Representative traces and the corresponding amplitude histograms are shown; c indicates closed channel state. Single channel currents were recorded with the patch-clamp technique in a symmetrical K^+ solution.

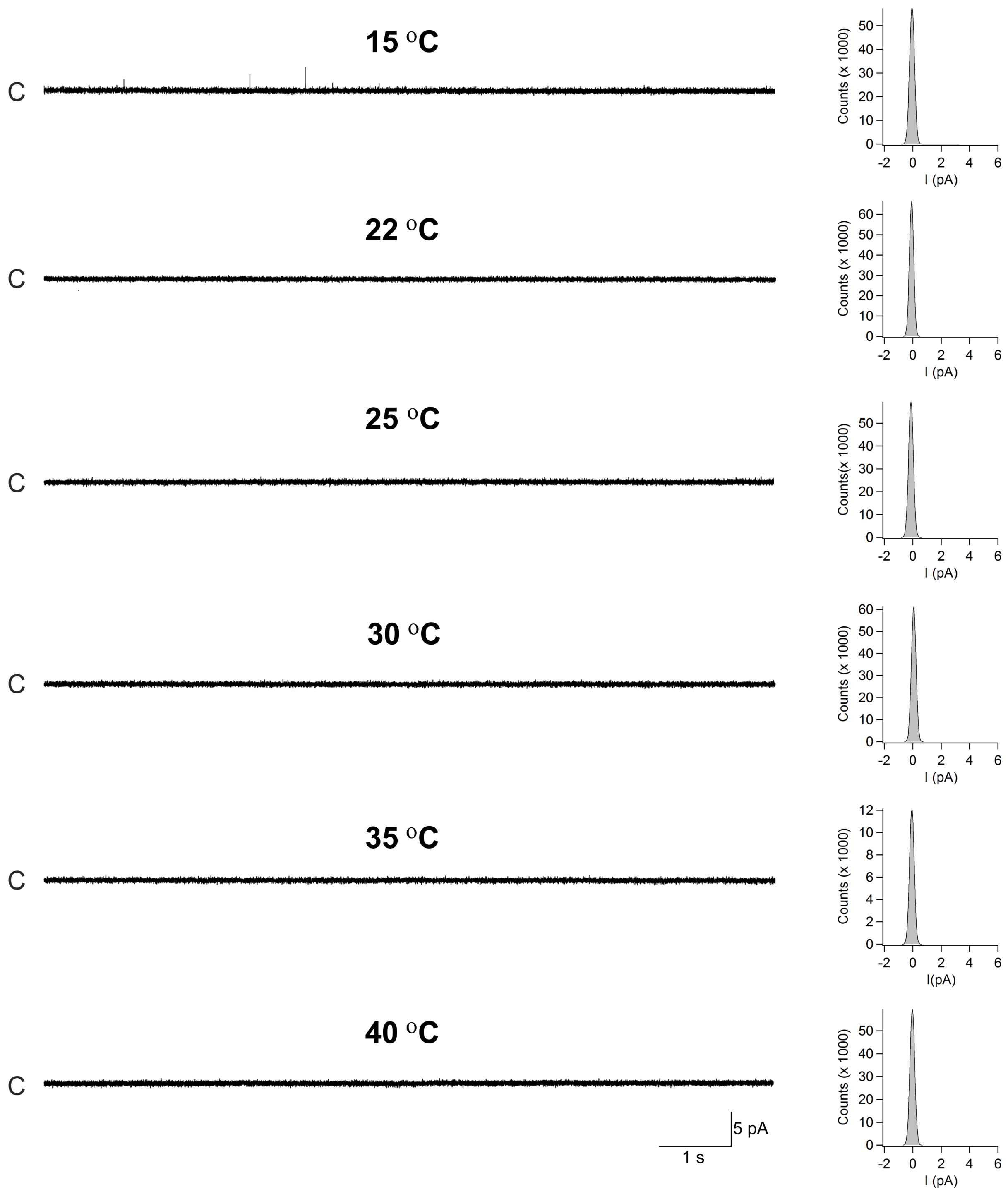

Supplementary Fig. 2. No currents were observed when lipid bilayers without hTRPA1 were exposed to various temperatures at a test potential of +60 mV (n = 3). Representative traces and the corresponding amplitude histograms are shown; c indicates closed channel state. Single channel currents were recorded with the patch-clamp technique in a symmetrical K^+ solution.

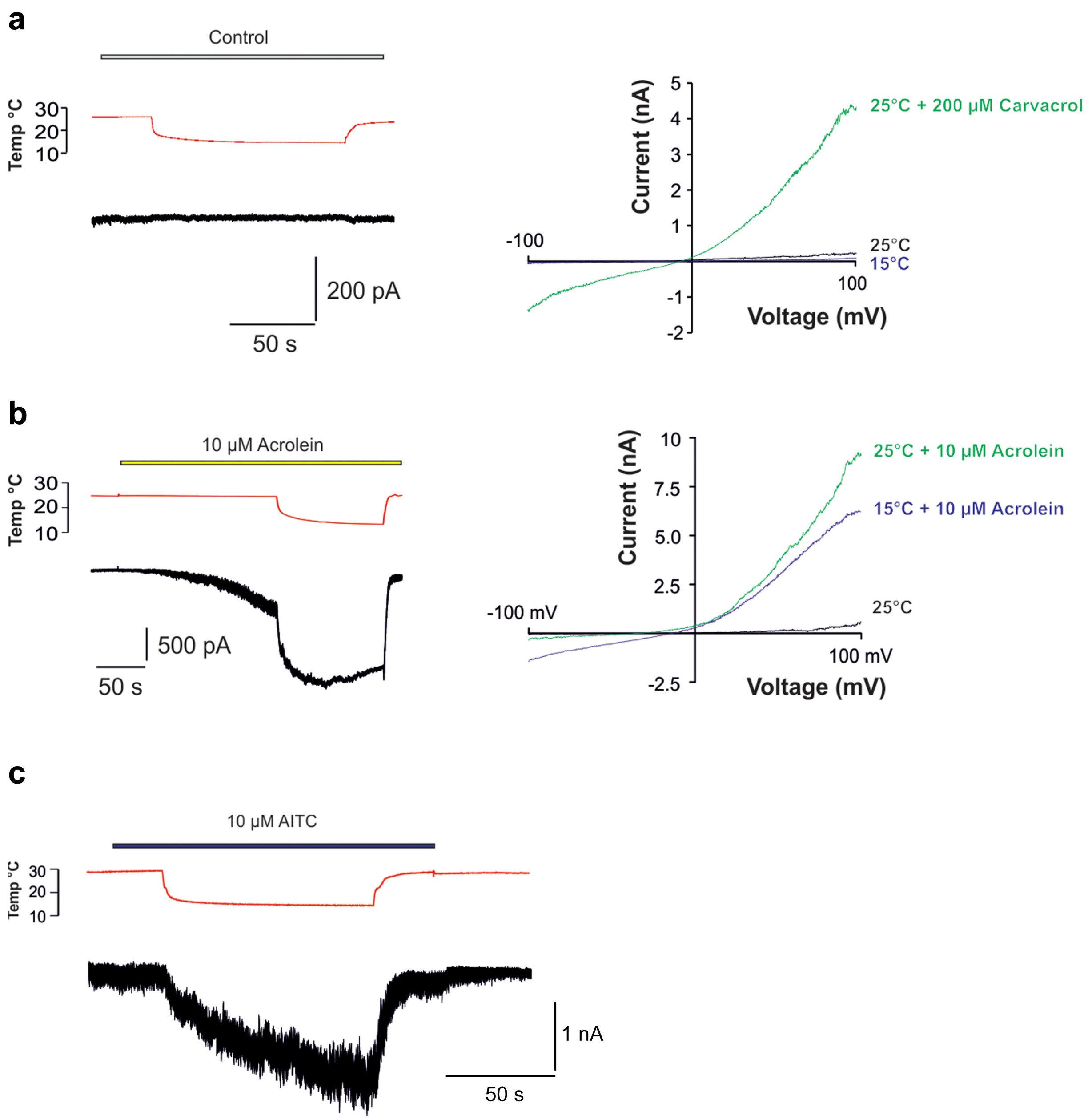

Supplementary Fig. 3. Representative traces showing the effect of cold in the absence and presence of electrophilic compounds on hTRPA1 expressed in HEK293t cells. (**a**) No inward currents were observed at 15 °C whereas the non-electrophilic compound carvacrol at a high concentration produced inward and outward currents confirming that hTRPA1 was functionally expressed. (**b** and **c**) The electrophilic compounds acrolein (n = 6-9) and allyl isothiocyanate (AITC, n = 4), at a concentration that produced no or minor hTRPA1 inward currents at 25 °C, triggered the cold-sensitivity of hTRPA1. Cells were either constantly held at a membrane potential of -60 mV (left panel traces) or subjected to 500 ms voltage ramps from -100 to + 100 mV (right panel traces).

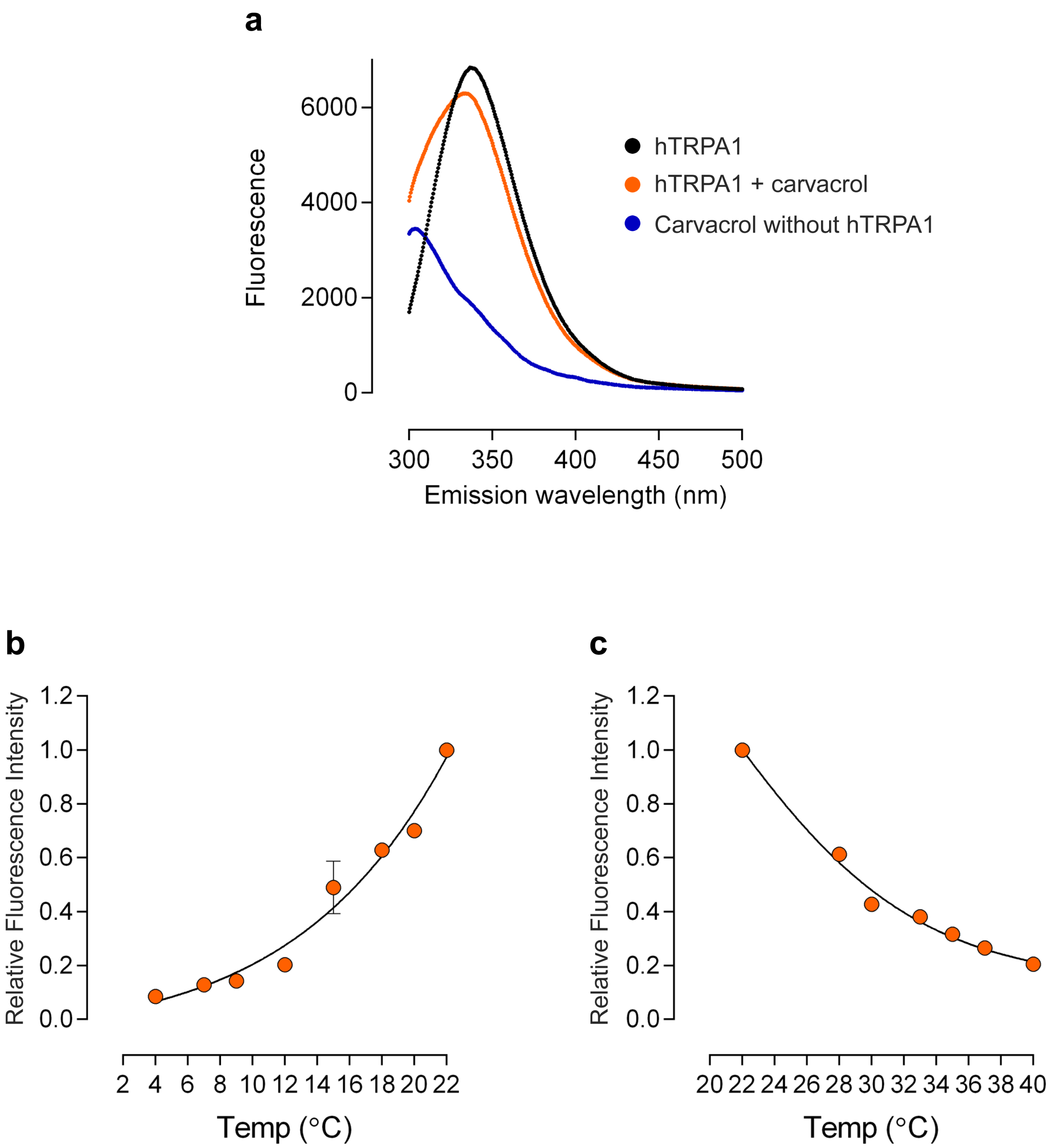

Supplementary Fig. 4. Representative fluorescence spectra showing the effect of the non-electrophilic compound carvacrol (100 μM) on hTRPA1 cold and heat responses. (**a**) At 22 °C, carvacrol itself emitted fluorescence that was subtracted when its effect on (**b**) cold and (**c**) heat was analyzed at the emission wavelength of 335 nm. The fluorescence intensity for each indicated temperature was related to that of 22 °C and expressed as Relative Fluorescence Intensity. Excitation was done at 280 nm and spectra were collected from 300 nm to 500 nm. Data are represented as mean ± s.e.m. of 3 separate experiments.

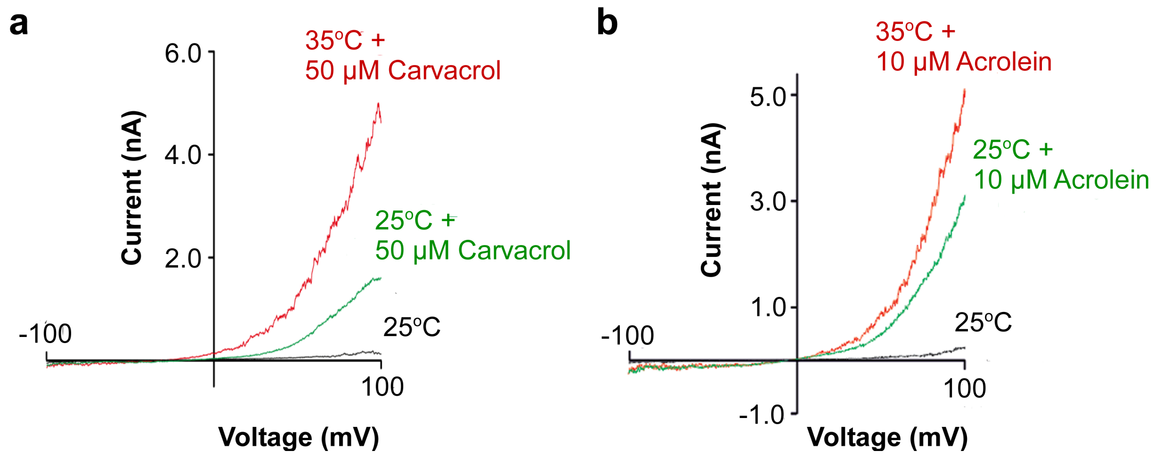

Supplementary Fig. 5. Representative traces showing the effect of the non-electrophile carvacrol and the electrophilic compound acrolein on hTRPA1 heat responses in HEK293t cells expressing hTRPA1. Heat currents in the presence of (**a**) carvacrol (n = 7) and (**b**) acrolein (n = 6) in cells subjected to 500 ms voltage ramps from -100 to + 100 mV.

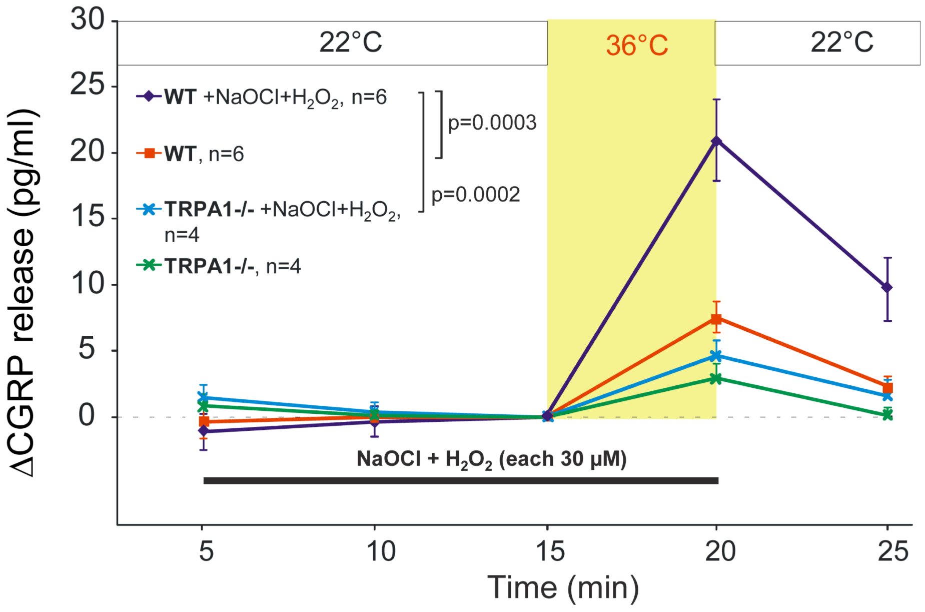

Supplementary Fig. 6. Heat-induced TRPA1-dependent neuropeptide release from mouse trachea. Shown is the experimental design for studying the effect of heat under various conditions on the release of the neuropeptide calcitonin gene-related peptide (CGRP) from mouse trachea as presented in **Fig. 6a**. Importantly, the combination of H_2O_2 and NaOCl, used to oxidize the cellular TRPA1 environment, did not cause CGRP release at the pre-incubation temperature of 22 °C. The release of CGRP is calculated as increase of CGRP over baseline (ΔCGRP in pg/ml) and data are represented as mean ± s.e.m. of separate experiments (n) as indicated in the graph. P values below 0.05 indicate statistically significant differences using ANOVA Tukey's honest significant difference test.
